# Monolithic Hybrid Abutment Crowns (Screw‐Retained) Versus Monolithic Hybrid Abutments With Monolithic Crowns (Adhesively Cemented): Three‐Year Data of a Prospective Clinical Split‐Mouth Study

**DOI:** 10.1111/jerd.13335

**Published:** 2024-10-26

**Authors:** Michael Naumann, Arndt Happe, Agnes Holtkamp, Sarah M. Blender

**Affiliations:** ^1^ Department of Prosthodontics, Geriatric Dentistry and Craniomandibular Disorders Charité – Universitätsmedizin Berlin Berlin Germany; ^2^ Private Practice Stahnsdorf Germany; ^3^ Center of Dentistry, Department of Prosthetic Dentistry Ulm University Hospital Ulm Germany; ^4^ Private Practice, Dr. Happe&Kollegen Münster Germany

**Keywords:** cemented, hybrid abutment, hybrid‐abutment crown, monolithic lithium disilicate, screw retained, split‐mouth design, titanium base

## Abstract

**Objectives:**

This study compares the restoration of single‐tooth implants with screw‐retained lithium‐disilicate hybrid‐abutment crowns and single‐tooth lithium‐disilicate crowns adhesively bonded to hybrid abutments with regard to objective clinical and subjective patient‐specific evaluation criteria over a time of observation of 3 years.

**Materials and Methods:**

Two bone‐level implants were placed in contralateral sides of the same jaw in 10 patients, each with two single‐tooth gaps. After osseointegration, implants were uncovered and an impression was taken. In accordance with the split‐mouth design, one implant in each patient was restored with a screw‐retained hybrid abutment crown and the other implant with a hybrid abutment and an adhesively bonded single‐tooth crown. The restorations were randomly allocated to the implants. Prefabricated titanium bases were used. The ceramic abutments and restorations were fabricated monolithically with pressed lithium‐disilicate ceramic. An objective evaluation (survival, technical, or biological complications, FIPS) by the practitioner and a subjective evaluation (satisfaction, OHIP) by the patient were carried out after 3, 6, 12, 24, and 36 months after restoration placement.

**Results:**

Both restoration types showed a survival rate of 100% after 3 years of observation. No technical or biological complications occurred. No significant difference was observed between the two types of restoration neither for objective (survival, technical or biological complications, FIPS) nor subjective (satisfaction, OHIP) evaluation criteria (*p* > 0.05).

**Conclusion:**

No statistically significant differences were observed between screw‐retained and cemented pressed lithium‐disilicate restorations on bone‐level implants for both objective and subjective evaluation criteria, respectively.

**Clinical Significance:**

Monolithic hybrid‐abutment crowns (screw‐retained) and monolithic hybrid abutments with single‐tooth crowns (cemented) made of pressed lithium disilicate can be used to successfully restore single implants.

## 1 Introduction

Hybrid abutment crowns and hybrid abutments restored with single‐tooth crowns offer an optimal alternative to the traditional monolithic abutments made of titanium or ceramic and combine the advantages of both materials [1].

Besides high mechanical stability, monolithic titanium abutments are highly biocompatible with the surrounding tissue [2, 3]. However, a disadvantage is their poor esthetic properties, which are especially problematic in esthetic challenging anterior areas, within gingival biotypes or in case of soft tissue recession [4, 5].

Ceramic materials, such as zirconium dioxide, show considerable esthetic advantages in this context and are also highly biocompatible with the intraoral structures [3, 6]. While oxide ceramics demonstrate high mechanical stability, the high hardness and brittleness of ceramic materials sometimes lead to undesired fractures of the material [7]. In addition, the high hardness of ceramic abutment materials in the direct restoration of titanium implants leads to wear of material in the implant‐abutment interface [8, 9]. The weakening of the connection and an increasing microgap between the implant and the superstructure can lead to technical and biological side effects. Due to the weakening of the connection and an increase in the microgap between the implant and the superstructure, technical, and biological side effects may occur [10, 11].

To avoid this problem, titanium bases can be used, which not only provide mechanically stability, but also ensure a homogeneous connection of the superstructure to the implant from a single material. The esthetic requirements, particularly in esthetically challenging regions, are then by the bonded ceramic abutments in the form of single‐tooth crowns or abutment superstructures [1].

A further benefit of these hybrid superstructures is their individual design in the area of the transmucosal emergence profile as a connection between the osseointegrated implant in the bone and the clinical visible, mostly supragingival restoration. The patient‐specific design is based on biological aspects to avoid peri‐implant associated diseases [12, 13] as well as esthetic aspects for the more natural design, shaping, and support of the soft tissue according to criteria, which follow the natural anatomical situation [14, 15].

Hybrid abutment crowns are monolithic single‐tooth crowns that are fixed extraorally on titanium bases. They are screwed intraoral direct onto the implant through an occlusal access, which is sealed afterwards with a composite material [1]. This type of restoration offers the advantage of no intraoral luting and therefore no excess luting material remains in the sulcus. In addition, these restorations can be used in regions with reduced vertical space, where no sufficient retention form for cementation would be achievable [16, 17, 18].

Hybrid abutments are custom‐made ceramic abutments in the form of individual abutments that are bonded extraorally to the titanium bases. Intraoral, this hybrid abutment is screwed onto the implant. In a second step, a customized single‐tooth crown is cemented onto the already screwed‐in hybrid abutment [19]. The additional abutment component allows even unfavorably angulated implant positions to be compensated for a later prosthetic restoration, which is especially important in the anterior region [16, 18, 20]. Due to the entire integrity of the restoration, no direct composite seal is visible and the restorations are more stable [21, 22].

In addition to high‐performance oxide ceramics, lithium disilicate ceramics can also be used for the fabrication of restorations and abutment suprastructures [23, 24]. Available for both the press technique and the CAD/CAM technique, this ceramic also demonstrates high tensile strength despite its high translucency and the resulting improved esthetics compared to zirconium dioxide [25], which is why it can be used on teeth and implants as part of single restorations in both the anterior and posterior region and has similar survival rates to other restorative materials [26, 27].

While lithium disilicate as a material for hybrid superstructure shows comparative mechanical properties under in vitro conditions compared to other established abutment materials [28, 29, 30] and it also shows promising survival rates in clinical studies with a short observation period, long‐term clinical observations are still needed [31, 32, 33, 34].

There is no clear advantage of screw‐retained or cemented restorations in the literature. While screw‐retained restorations are more likely to be associated with technical problems such as chipping of the ceramic restorations or loosening or fractures of the abutment screws, cemented restorations show more biological complications such as inflammation of the peri‐implant tissue due to excess and retained luting material in the peri‐implant sulcus [35, 36, 37]. Regardless of the fixation of the restorations, the most common technical complication reported for both screw‐retained and cement‐retained restorations is the loosening of the abutment screw [36, 38, 39, 40]. Screw‐retained restorations have a considerable advantage in this context as they are associated with simple and fast retrievability due to quick access to the abutment screw and its replacement [35, 41]. Cemented restorations, on the other hand, are very challenging, as the exchange of the screw is associated with the trepanation or removal of the crown. If the exact position of the screw channel is not documented, this can involve a huge amount of effort and unnecessary invasiveness, which in the worst case can result in a replacement of the restoration [38, 42].

In addition to the indications and advantages of the different therapy alternatives, an objective or subjective assessment by dentists and patients can therefore be used to select the right therapy. For the objective evaluation of (implant) prosthetic restorations, scores are available in addition to the determination of survival rates and complication rates, which allow an evaluation of the prosthesis depending on defined criteria and their characteristics. The functional implant prosthodontic score (FIPS) applied in this study was presented by Joda et al. and provides an objective and reliable evaluation of single‐tooth implant restorations based on clinical and radiographic results [43, 44].

The subjective evaluation of the restoration by the patient provides a quantitative measurement of patient satisfaction before and after treatment using visual analogue scales literature, as well as the determination of the influence of treatment on oral health‐related quality of life using standardized patient evaluation sheets. For this purpose, the Oral Health Impact Profile is used, a questionnaire that is used worldwide to assess oral health‐related quality of life and is thus highly researched [45]. Patient‐reported outcome measures (PROMs) allow patients to assess the functional, social, and psychological effects of oral treatment. While in the past these aspects played a much less important role in the assessment of therapy options, today they are given great importance [46]. Patient‐related assessments should be used in all clinical studies to document the outcome of oral rehabilitation with implants and thus represent an important and crucial component in the patient‐specific decision‐making process for various therapy alternatives [47].

Taking the above aspects into account, this prospective, randomized clinical pilot study aimed to evaluate the performance of (A) monolithic hybrid abutment crowns made of lithium disilicate and (B) monolithic hybrid abutments and adhesively cemented single‐tooth crowns made of lithium disilicate regarding objective and subjective evaluation criteria after 3 years of clinical observation.

The following null hypotheses were formulated.

- There are no differences in the objective evaluation criteria between the two study groups: 3‐year survival rate, complication rate regarding technical and biological criteria and Functional Implant Prosthodontics Score (FIPS)
- There are no differences in the subjective evaluation criteria between the two study groups: Patient satisfaction and the measured oral health‐related quality of life (OHIP).

## 2 Materials and Methods

### 2.1 Study Design

The present study is a prospective, randomized clinical pilot study in a split‐mouth design that was performed monocentrically under practice conditions. The study was registered prior to the start at the German Register of Clinical Studies (Freiburg; DRKS‐ID: DRKS00005680) and the study design was approved by the Institutional Review Boards of the University of Ulm (approval number 193/13, 2013‐07‐29). Written informed consent was requested from all patients recruited for the study prior to possible inclusion. The investigations were performed in accordance with the World Medical Association Declaration of Helsinki, the German Medical Devices Act, ISO14155 Clinical investigation of medical devices and good clinical practice.

### 2.2 Study Protocol, Inclusion, and Exclusion Criteria

In the present study, a total of 10 patients (*n* = 10) were treated, each of them was restored with two implants on contralateral sides in the same jaw. The position of the implants in the dental arch was the same in both sides of the jaw from the midline. Table 1 provides a detailed overview of the defined inclusion and exclusion criteria of the study.

**TABLE 1:** Overview of the defined inclusion and exclusion criteria of the study.

Inclusion criteria	Exclusion criteria
– Patients older than 18 years, legally competent – Written informed consent is given prior to the beginning of the study – Completed periodontal and Conservative pre‐treatment – Single tooth gaps on contralateral sides in the same jaw that have to be restored with implant prosthetic restoration – Gap width > 7 mm – Sufficient crestal bone supply, implantations without need for augmentation – Antagonists existing in the area of the new prosthetic restorations – Both implants are compatible with both prosthetic treatment options	– No compliance – Untreated, acute periodontitis – Poor oral hygiene – Untreated bruxism – Pregnancy – Nicotine consumption – Alcohol or drug abuse – Infectious disease (hepatitis/HIV/AIDS) – Severe diabetes mellitus, insulin use

**FIGURE 1:**
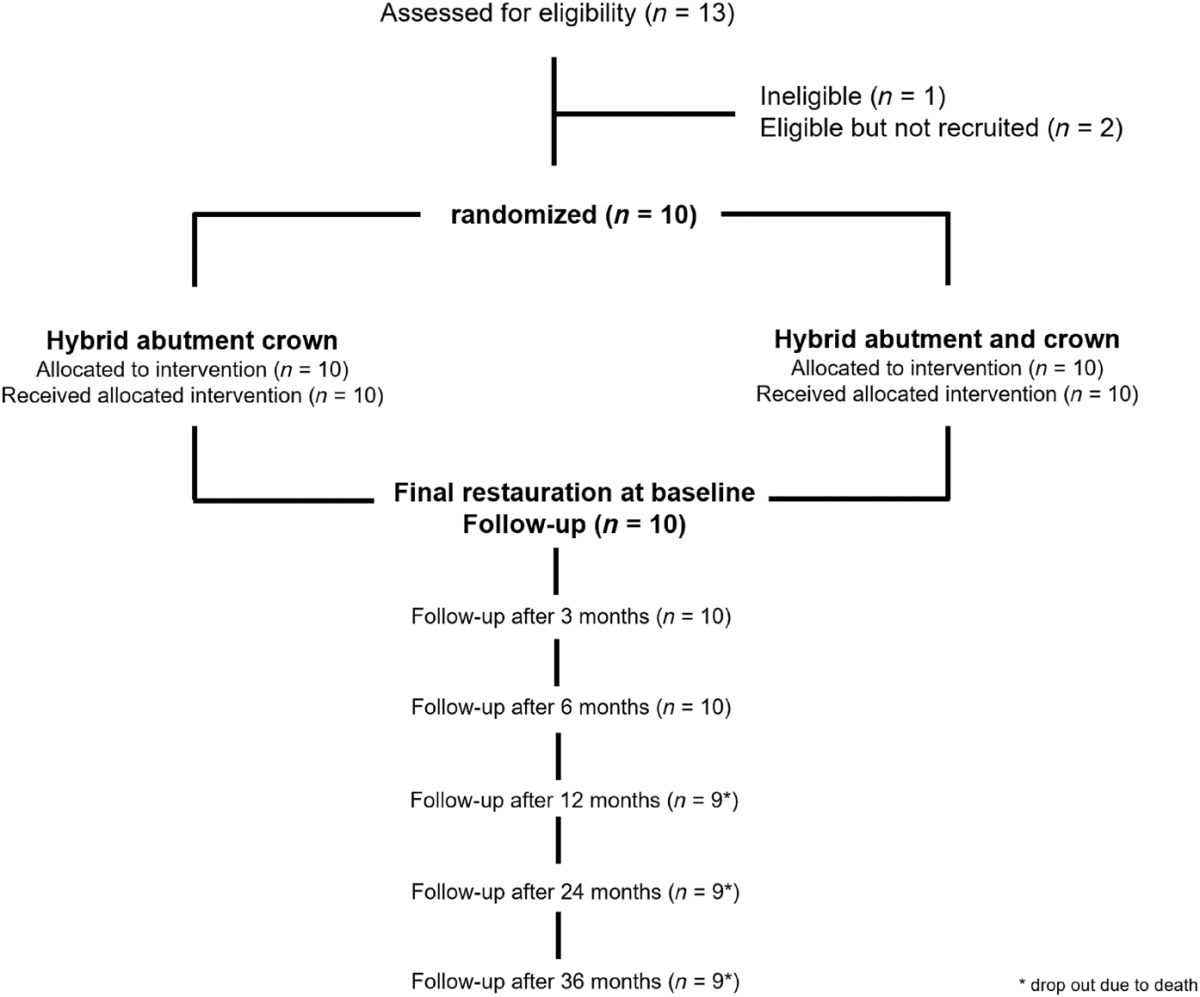
Flow diagram of participants according to study design and to the CONSORT statement.

Over a period of 6 months, a total of 13 patients were screened according to the inclusion and exclusion criteria listed above, of which one patient was not accepted due to the inclusion and exclusion criteria, and two other patients were not recruited for the study (Figure 1).

**FIGURE 2:**
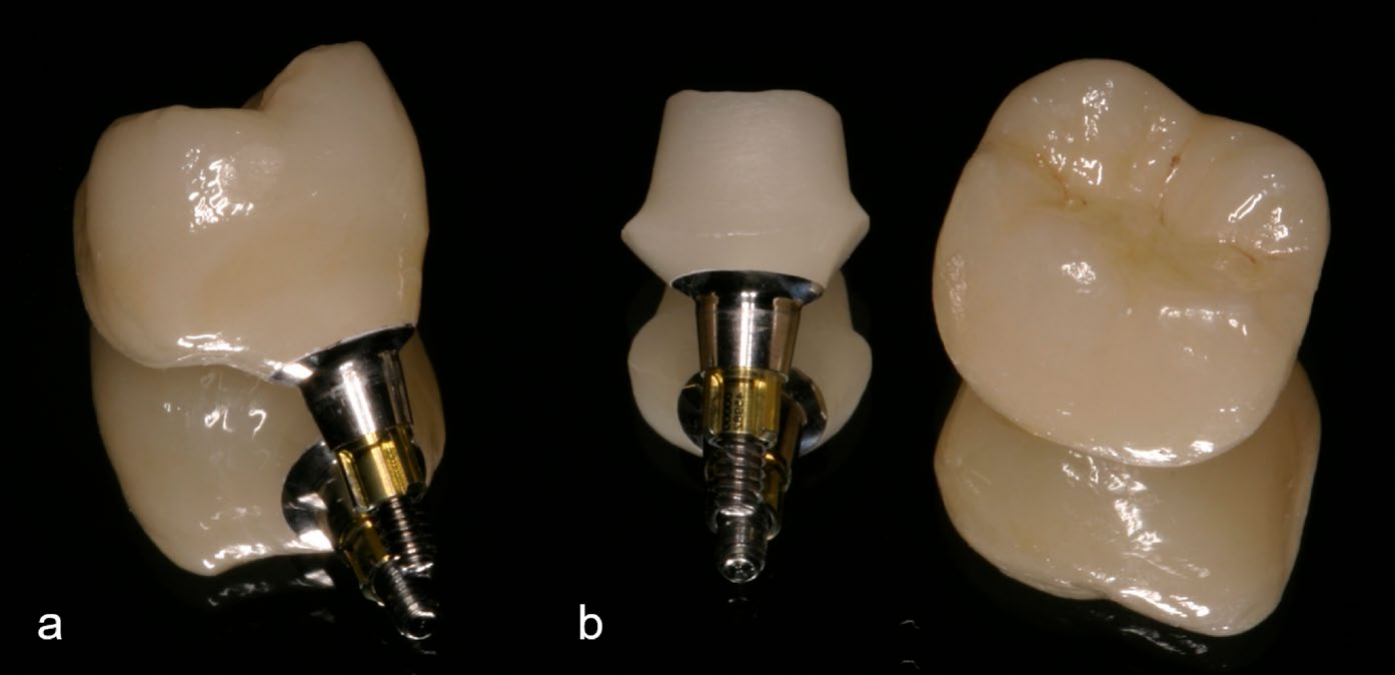
The types of restorations investigated in the study: (a) hybrid abutment crown consisting of a titanium base and monolithic lithium disilicate single crown after extraoral bonding; (b) hybrid abutment consisting of a titanium base and monolithic abutment superstructure and monolithic single crown, both made of lithium disilicate.

Using the split‐mouth study design, the implants of each patient were restored with the two therapy options: (A) hybrid abutment crowns made of lithium disilicate (Figure 2a) and (B) hybrid abutment and adhesively cemented single‐tooth crowns made of lithium disilicate (Figure 2b).

The randomization of which implant received which type of prosthetic restoration was randomized based on randomization envelopes and on a randomization list prepared by the Department of Biostatics, University of Ulm, Ulm, Germany.

**FIGURE 3:**
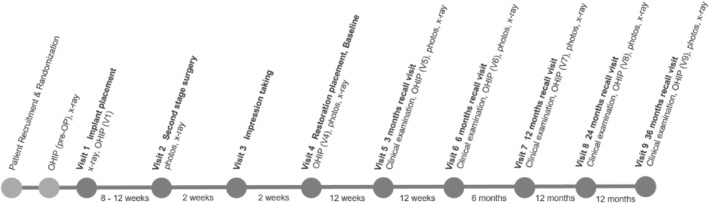
Overview of the individual study visits in the presented study.

Figure 3 provides an overview of the individual study dates and the procedures performed.

As part of the preoperative preparation, x‐rays of the respective implantation area were taken for all patients and an OHIP assessment form was completed.

All surgical and prosthetic examinations were performed by a single dentist (author M.N.) to ensure standardized treatment of all patients.

### 2.3 Surgical Procedure

Ten patients received two implants in the same position in the dental arch on the two opposite sides of one jaw at the time of implant placement (Visit 1) (CONELOG SCREW‐Line implants; Camlog Biotechnologies AG, Basel, Switzerland, conical implants with in internal conic connection, beveled implant shoulder angle 45° platform switching). The length and diameter of the implants were selected according to the patient's individual anatomical situation. No bone augmentation was performed. Before implant placement, the patients rinsed with a 0.12% CHX mouth rinse solution. The implantation was done under local anesthesia and was performed according to the drilling protocol indicated by the implant manufacturer. A subsequent control x‐ray was taken to check the implant position. The implants were then covered with cover screws and were left to heal in closed position. The patients were informed postoperatively about the use of a 0.1% CHX mouth rinse solution twice daily and careful cleaning of the teeth.

**FIGURE 4:**
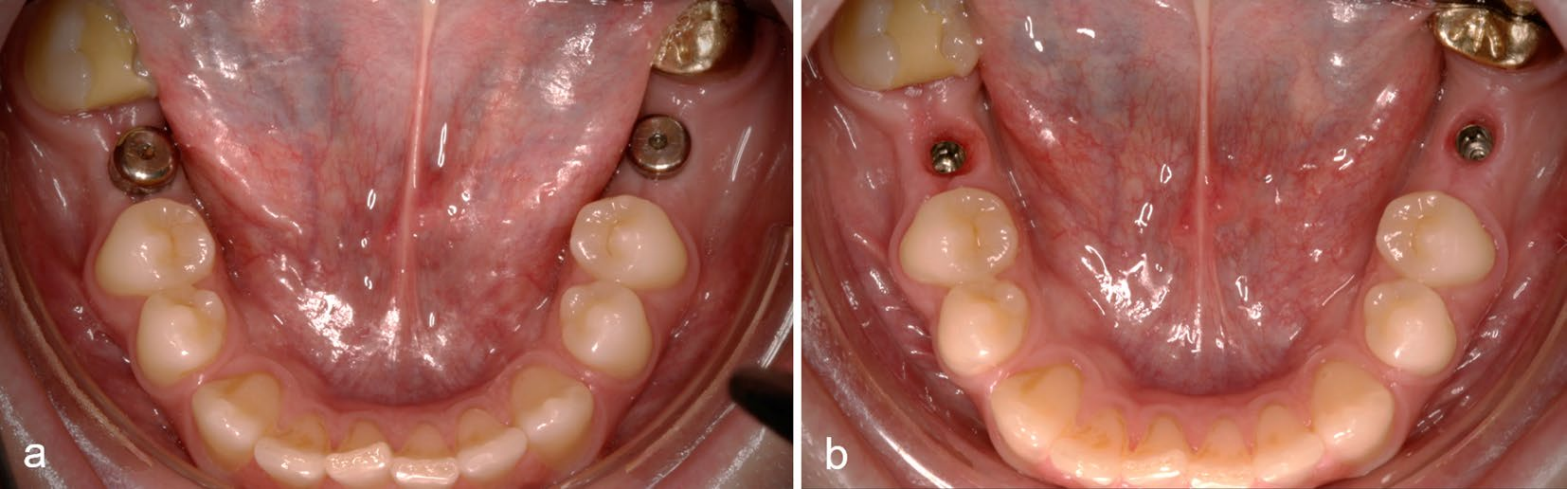
Clinical documentation of a patient case from the study. (a) Clinical situation after uncovering the implants with healing abutments in regions 36 and 46; (b) clinical situation 2 weeks after uncovering the implants and forming the emergence profile. The gingiva formers were removed in order to take the impression of the implants.

The implants were exposed with gingiva formers after 3 months in the upper jaw and after 2 months in the lower jaw (Visit 2) (Figure 4a). Additional photos and a control x‐ray of the uncovered implants were taken.

### 2.4 Second Stage Procedure and Prosthesis Placement

The impression of the implants was taken 10–14 days after uncovery (Visit 3; Figure 4b). During this time, the implants were restored with gingiva formers to shape an esthetic emergence profile. Impression posts were screwed onto the bone‐level implants for impression taking of the implants using the closed tray technique and repositioning aids were placed on them to enable exact repositioning of the impression posts and the laboratory analogs in the impression. The impression was taken with individual impression trays and a single‐phase, A‐silicone‐based precision impression material (Aquasil Ultra Monophase, Corp. Dentsply, USA). After impression taking, the internal threads of the implants were cleaned with 0.12% CHX, the interior of the implants were dried with paper points and the implants were then restored with healing abutments. A facebow was then created for individualized fixation of the models in the articulator and a bite registration was made in centric occlusion. In addition, the patient's individual tooth shade was documented. X‐ray images and intraoral photo documentation were taken for later evaluation to objectively assess the appearance of implant‐supported restorations in combination with the surrounding hard and soft tissue according to the criteria of the FIPS.

The impressions were then sent to an external dental laboratory (Biemadent Zahntechnik Berlin, Germany), gypsum models with laboratory analogs and gingival masks in the area of the implants were manufactured and fixed in the articulator in a patient‐specific manner. Due to the split‐mouth design of the study, one implant in each patient was restored with an (A) hybrid abutment crown made of lithium disilicate and a (B) hybrid abutment and an adhesively bonded single‐tooth crown made of lithium disilicate.

The restorations were fabricated strictly in accordance to the manufacturer's instructions for the materials used. The same prefabricated titanium bases were used for both types of prosthetic restoration. The ceramic hybrid abutment crowns and the hybrid abutments were waxed up on the titanium bases according to the patient's individual emergence profile and tooth shape, embedded and then fabricated from pressable IPS e.max lithium silicate blanks according to the minimum layer thicknesses specified by the manufacturer. In order to ensure uniform fabrication of the restorations, the same dental technician, who had previously been trained in the fabrication of implant superstructures at a training center of the ceramic manufacturer Ivoclar Vivadent, Liechtenstein, performed the dental technical work steps in every case in the same way.

The fabricated hybrid abutment crowns and hybrid abutments were then cemented extraoral to the prefabricated titanium bases using a self‐curing adhesive luting material (Multilink Implant, Corp. Ivoclar Vivadent, Liechtenstein) according to the manufacturer's instructions. The titanium bases were circular sandblasted (aluminium oxide, 50 μm, 2 bar) in the area of the bonding surface, cleaned, dried with oil‐free air, and finally the bonding surface was silanized for 60 s (Monobond Plus, Ivoclar vivadent, Liechtenstein). For bonding, the titanium base was then screwed onto the model and the screw channel was covered with foam pellets to protect the screw from the luting material. The inner surfaces of the ceramic restorations were conditioned with IPS Ceramic Etching Gel for 20 s, cleaned, dried with oil‐free air, and finally silanized for 60 s (Monobond Plus, Ivoclar vivadent, Liechtenstein). The adhesive luting material was then applied to the vertical walls of the crown lumen, the restoration was placed on the titanium bases in the correct position using finger pressure and excess luting material was removed. Finally, the cementation lines were sealed with a glycerine gel (Liquid Strip, Ivoclar Vivadent, Liechtenstein) to ensure complete auto‐polymerization of the material. After complete curing, material residues of the glycerine gel and the luting material were removed and the cementation lines were finished with ceramic and resin polishers.

On the day of placement of the restorations (Visit 4, baseline), the implant superstructures were clinically tried in and evaluated according to clinical parameters for the insertion of restorations. For final cementation, the superstructures were screwed onto the implants intraorally with a torque of 20 N cm. The occlusally visible screw channel was filled with Teflon tape and composite for both the hybrid abutment crown and the hybrid abutment (IPS Empress Direct, Corp. Ivoclar Vivadent, Liechtenstein). The composite seal of the hybrid abutment crown was then polished to check and adjust the static and dynamic occlusion.

**FIGURE 5:**
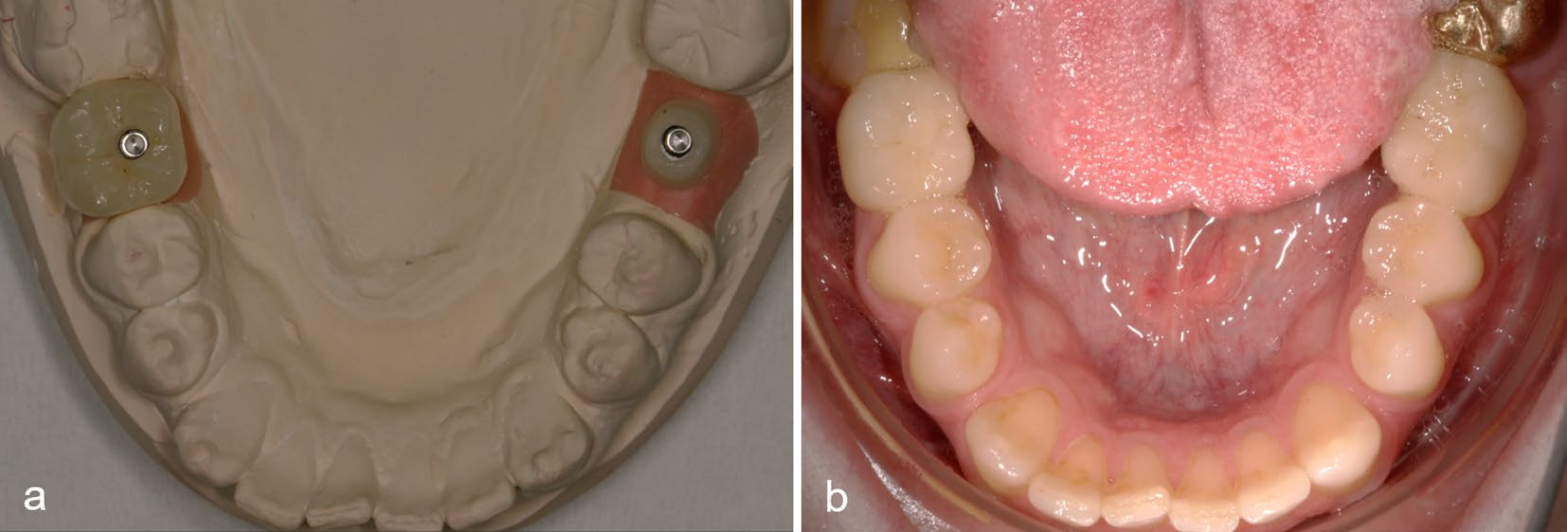
Presentation of the fabricated superstructures: (a) Model situation of the fabricated restorations. The implant 46 is restored with a monolithic hybrid abutment crown made of lithium disilicate, which was bonded extraorally onto the titanium base. The occlusal screw access is visible. Implant 36 is restored with a monolithic hybrid abutment, which is bonded intraorally with a monolithic single crown made of lithium disilicate (the crown is not visible in this image); (b) clinical situation of the inserted restorations. The occlusal screw channel of the hybrid abutment crown on implant 46 was directly closed with a tooth‐colored composite.

The already inserted hybrid abutments were then covered with the single‐tooth crowns made of lithium disilicate. For this purpose, the restorations were cemented with a self‐adhesive and chemically curing luting material (SpeedCem, Ivoclar Vivadent, Liechtenstein). The inner surfaces of the restorations were prepared in the same way as the usual pre‐treatment of lithium disilicate as described above (Figure 5).

**FIGURE 6:**
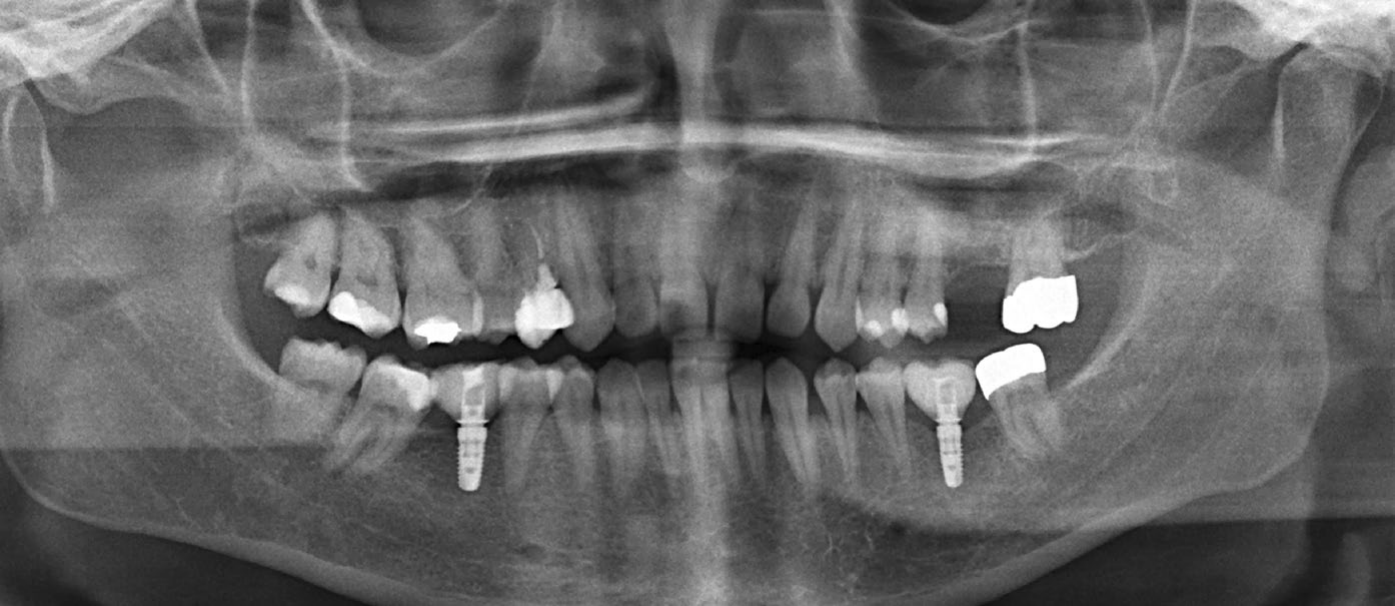
Final x‐ray to evaluate the hard tissue around the implants. Implant 46 is restored with a monolithic hybrid abutment crown made of lithium disilicate; implant 36 is restored with a monolithic hybrid abutment and a monolithic single crown made of lithium disilicate.

In addition to taking intraoral photographs and an X‐ray (Figure 6), the OHIP sheet was filled out again after incorporation.

### 2.5 Follow‐Up Examinations

The follow‐up examinations of the implants and restorations were carried out 3, 6, 12, 24, and 36 months after placement of the implant suprastructures.

The following observations were made on all teeth:

– Clinical examination, consisting of measuring the 4‐point probing depths (mesial, buccal, distal, oral), the approximal plaque index (API), the papilla bleeding index (PBI) and the periodontal screening index (PSI)
– OHIP
– Intraoral photographs

At the follow‐up examination 36 months after restoration, additional x‐rays were taken to determine the FIPS.

In addition to the documentation of the described findings, a professional tooth cleaning with oral hygiene instructions was carried out at each follow‐up appointment.

### 2.6 Study Outcome Measures

When comparing the two therapeutic interventions, the study outcome measures can be divided into objective (survival and complications, clinical examination, FIPS), and subjective (patient satisfaction, OHIP) evaluations. Objective evaluations are performed by the practitioner or dentist using defined criteria and scores. Subjective evaluations were carried out by the patient.

Pre‐defined indices (ST, API, PBI, and PSI) were recorded as part of the clinical examinations. Pre‐defined biological and technical complications were recorded to determine the survival and complication rate over time. If such complications occurred during the follow‐up examination, they were documented.

### 2.7 Functional Implant Prosthodontic Score

The FIPS is a score for the clinical and objective evaluation of implant‐supported single‐tooth restorations. For this purpose, the restorations are rated based on five standardized and objective variables (Table 2).

**TABLE 2:** Definition of the functional implant prosthodontic score [43, 44].

Variables	0 points = major discrepancy	1 point = minor discrepancy	2 points = no discrepancy
Interproximal contacts and papillae	Both, contact and papillae incomplete	One incomplete	Ideal: papillary conditions and appearance, mesial & distal contact areas
Occlusion Static and dynamic	Supra‐contact	Infra‐contact	Ideal: light occlusal contacts without dynamic interactions
Design Contour and color	Contour plus color deficiencies	Solely color deviations	Harmonious crown matching to the individual patient situation
Mucosa Quality and quantity	Nonkeratinized + non‐attached	Nonkeratinized + attached	Keratinized + attached
Bone X‐ray	Radiographic bone loss > 1.5 mm	Radiographic bone loss < 1.5 mm	No radiographic bone loss
Maximum score			10

*Note*: Each variable can be rated with a value of 0–2 (0 = major discrepancy, 1 = minor discrepancy, 2 = no discrepancy), which allows a maximum score of 10 per restoration. The evaluation is based on a clinical examination and the use of current intraoral photographs and x‐rays (from Visit 9).

In the present study, the clinical examination and evaluation were carried out independently by two dentists with at least 10 years of professional experience in implant dentistry.

### 2.8 Patient Satisfaction

To document subjective patient satisfaction, patients were asked to document their subjective satisfaction with the appearance of the implant crowns on a visual analog scale. For this purpose, a visual analog scale from 1 to 10 (1 = worst assessment, 10 = best assessment) was used. The rating took place both before the implants were restored and at all follow‐up visits.

### 2.9 Oral Health‐Related Quality of Life

The German version of the Oral Health Impact Profile (OHIP) with 53 questions was used to assess oral health‐related quality of life. In addition to the 49 original questions (OHIP‐43), this version also contains four additional questions in German (OHIP‐G53). The OHIP contains questions on how the patient's individual condition (in particular the intraoral situation, teeth, or dentures) affects the patient's overall state. The patient has five possible answers (very often, often, sometimes, rarely, never) which allow a maximum score of 196 points (OHIP‐43) or 212 points (OHIP‐G53).

The OHIP was completed directly at the beginning of Visit 1 and Visit 2 (no prosthetic restoration was inserted), as well as at all follow‐up times after restoration (Visit 5‐ Visit 9). No OHIP was completed at the examination times of the impression and the restoration of the implants.

### 2.10 Statistical Analysis

The descriptive and statistical analysis of the data obtained was performed using the following software: IBM SPSS Statistics V25 for Windows (Corp. IBM, Armonk, NY, USA). The significance level was set at *p* < 0.05. The inter‐rater reliability in relation to the evaluation of the FIPS was analyzed using Cohens‐Kappa statistics according to Landis and Koch. The statistical analysis of the OHIP evaluations was carried out using Box Whisker Plot and Box‐Cox transformation.

## 3 Results

The matched‐pairs sample comprises a total of 10 subjects who each received two different implant‐prosthetic restorations in one jaw on two opposite sides: (A) monolithic hybrid abutment crown (screw‐retained) and (B) hybrid abutment with adhesively bonded single‐tooth crown (adhesively cemented).

### 3.1 Characteristics of the Study Population

Six male (60%) and four female (40%) patients with an average age of 55 years (minimum 36 years, maximum 77 years) were enrolled. One drop‐off was documented after the 6‐month follow‐up because the patient died unexpectedly of cancer.

**FIGURE 7:**
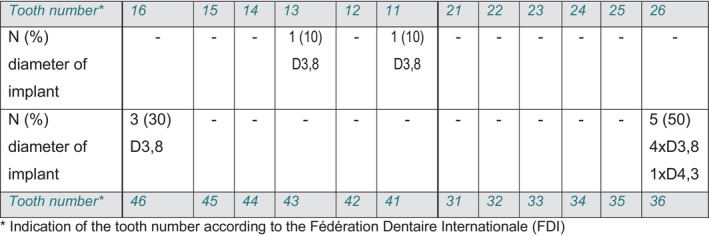
Overview of the inserted implants that were restored with a hybrid abutment crown (screw‐retained); indication of the position of the implants in the jaw by indicating the tooth number according to the FDI system, as well as the number (N) of implants in the individual positions as a percentage. The diameters of the inserted implants are also indicated.

**FIGURE 8:**
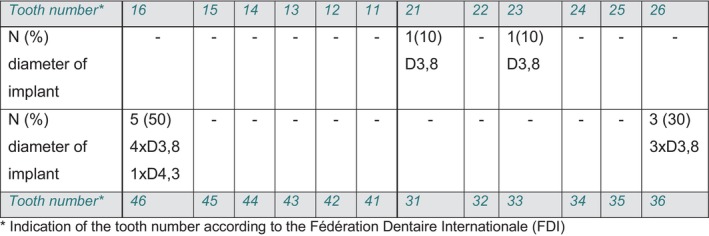
Overview of the inserted implants that were restored with a hybrid abutment and an adhesively fixed crown (cemented); indication of the position of the implants in the jaw by indicating the tooth number according to the FDI system, as well as the number (N) of implants in the individual positions as a percentage. The diameters of the inserted implants are also indicated.

In two patients, the implants were placed in the maxilla in the anterior region, while in eight patients all implants were placed in the mandibular first molar region. A detailed overview of the position of the implants in the jaw and the diameters used can be found in Figure 7 for the monolithic hybrid abutment crowns (screw‐retained) and in Figure 8 for the hybrid abutments with adhesive‐retained crowns (cemented).

The average treatment time for the impression taking of the two implants (Visit 3) was 65.7 min on average with a standard deviation of 38.6 min. The placement (Visit 4, baseline) of the two implant‐prosthetic restorations took an average of 73.2 min with a standard deviation of 25.2 min. A statistical comparison between the two implant‐prosthetic restorations is not possible in this case, as each patient received both restoration options and they were fabricated and delivered in parallel. It was not possible to clearly separate the placement of the two restoration options.

### 3.2 Comparison of Survival and Complications

After 36 months, both implants and both restorations (survival in both groups was 100%) were in situ for all patients (*n* = 9, one drop‐out due to death after 6‐month follow‐up). No technical or biological complications were documented during the entire observation period.

A statistical evaluation of the survival rates of the two treatment methods was not useful due to the lack of failures.

### 3.3 Comparison of the Indices Measured During the Clinical Examination

Table 3 provides an overview of the documented 4‐point measurements for pocket‐depth and the recorded indices such as PSI, API, and PBI at baseline and at the 36‐month follow‐up examination. No significant differences were observed between the two restoration groups.

**TABLE 3:** Comparison of the measured clinical indices of the matched‐pairs samples at the time of the baseline examination and the follow‐up examination after 36 months for the (A) hybrid abutment crowns (screw‐retained) and the (B) hybrid abutments with an adhesively luted crown (cemented); mm = millimeters; PSI = periodontal screening index; API = proximal plaque index; SBI = sulcus bleeding index; m = mesial; v = vestibular; d = distal; o = oral.

	Patient	Baseline							36 month recall (V9)						
		Pocket depth in mm							Pocket depth in mm						PBI
		*m*	*v*	*d*	*o*	PSI	API	PBI	*m*	*v*	*d*	*o*	PSI	API	
(A) Hybrid abutment crowns (screw‐retained)	1	3	2	2	1	2	1	0	1	1	1	1	2	0	0
	2	2	2	3	3	2	0	1	2	1	1	1	2	1	1
	3	2	4	1	1	3	1	1	3	2	4	2	3	1	1
	4	3	3	5	2	3	1	0	5	3	4	2	3	1	0
	5	2	3	3	3	2	1	1	5	3	4	2	3	1	0
	6	4	3	4	2	3	1	1	—	—	—	—	—	—	—
	7	—	—	—	—	2	—	1	3	2	3	2	2	1	1
	8	3	3	2	1	2	1	1	3	2	3	2	2	1	2
	9	4	2	2	1	3	0	0	3	2	3	3	1	1	0
	10	3	2	2	2	1	0	0	1	1	1	1	2	0	0
(B) Hybrid abutments with an adhesively luted crown (cemented)	1	2	2	2	1	2	1	0	2	1	2	2	2	0	0
	2	3	2	2	2	2	0	1	2	1	3	2	2	1	1
	3	3	5	3	2	3	1	1	5	4	5	4	3	1	1
	4	4	3	5	2	3	1	0	4	3	5	2	3	1	0
	5	6	1	3	2	3	1	1	4	3	4	2	3	0	0
	6	4	3	4	2	3	1	1	—	—	—	—	—	—	—
	7	—	—	—	—	2	—	1	3	3	3	2	2	1	1
	8	4	2	5	2	3	0	1	3	3	3	2	2	1	2
	9	3	3	3	3	1	0	0	4	2	2	3	3	0	0
	10	2	2	2	2	1	0	0	3	3	3	2	1	1	0

### 3.4 Evaluation of Functional Implant Prosthodontic Score (FIPS)

Table 4 provides an overview of the mean values (SD; Max; Min) of the evaluations of both examiners for the two restoration types.

**TABLE 4:** Mean values, standard deviation (SD), maximum and minimum FIPS scores of the two independent examiners for both restoration types.

	(A) Hybrid abutment crowns	(B) Hybrid abutments with an adhesively luted crown
Examiner 1	9.3 (SD 0.7; Max: 10, Min: 8)	8.4 (SD 1.2; Max: 10, Min: 6)
Examiner 2	9.0 (SD 0.7; Max: 10, Min: 8)	8.5 (SD 1.6; Max: 10, Min: 6)

The agreement between the two investigators was calculated using Cohen‐Kappa and amounted to *k* = 0.237 (sufficient agreement of the results).

### 3.5 Comparison of Patient Satisfaction

The data on the subjective satisfaction of the patients with the clinical appearance of the restorations before restoration of the implants and after 36 months after restoration are shown in Table 5.

**TABLE 5:** Documented subjective patient satisfaction before the implants were restored and after 36 months, indicating the documented values of the visual analog scale.

Values on VAS regarding subjective satisfaction	1	2	3	4	5	6	7	8	9	10
Number of values before restoration			1	2	6		1			
Number of values 36 months after restoration								1^a^		8

^a^ Patient complained about a gap in the molar region, no direct relation to the implant restoration.

Before restoration, this results in a mean value of 4.8 (SD 1.0; Max 7; Min 3), at 36 months after restoration 9.8 (SD 0.7; Max 8; Min 10).

### 3.6 Comparison of the Oral Health Impact Profile

**FIGURE 9:**
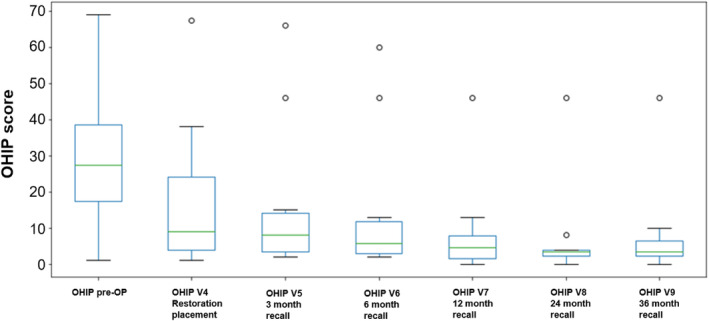
Box Whisker Plot of pooled OHIP scoring by patients per recall visit.

Figure 9 shows descriptively the measured OHIP values of all patients at the individual examination times (pre‐OP; Visit 5—Visit 9) using a box whisker plot.

Large standard deviations at visits 1 and 2 suggest that the OHIP questionnaires were not evaluated uniformly. During the progress of the study, the descriptive values became more uniform and the standard deviations smaller. The median of all visits ranges from 5 to 30.

The statistical analysis of the questions (OHIP) shows no statistically significant differences with regard to the factors age (OHIP G‐53: *p* = 0.717; OHIP—49: *p* = 0.760) and gender (OHIP G‐53: *p* = 0.366; OHIP—49: *p* = 0.192).

**FIGURE 10:**
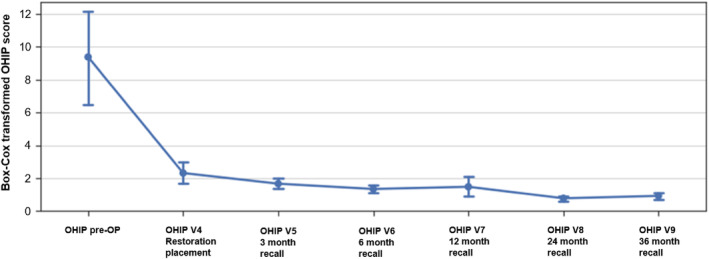
Box‐Cox transformed Score (CI 95%) of OHIP scoring, pooled data for both groups per recall visit.

**FIGURE 11:**
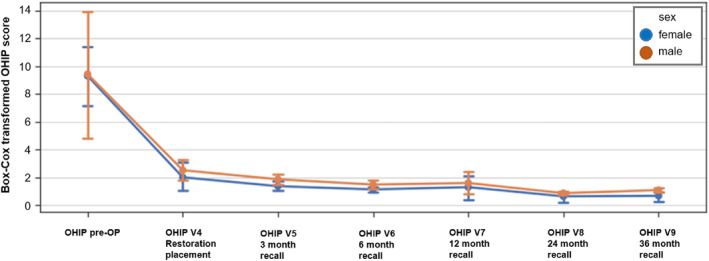
Box‐Cox transformed Score (CI 95%) of OHIP scoring per recall visit distinguished by gender (blue = female; orange = male).

**FIGURE 12:**
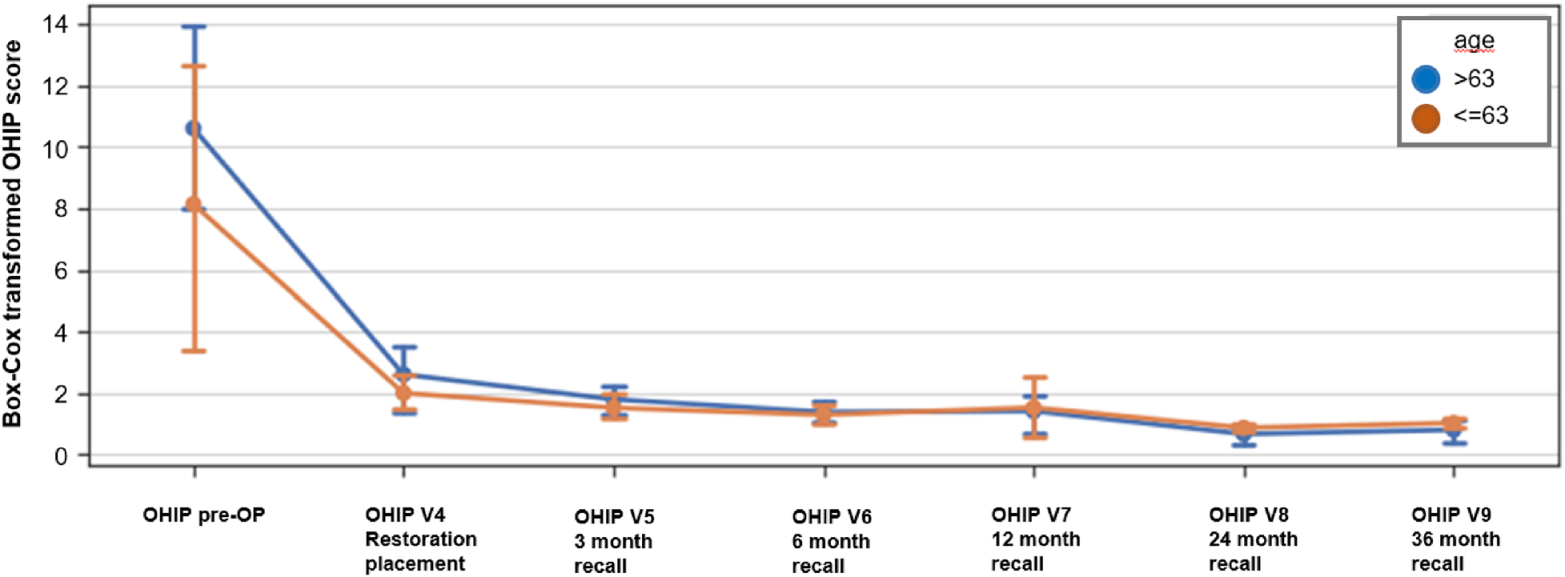
Box‐Cox transformed Score (CI 95%) of OHIP scoring per recall visit distinguished by age (blue = age > 63; orange = age < = 63).

The factor screw‐retained or cemented restoration could not be analyzed, as all patients had both restorations in situ and did not fill out separate questionnaires (Figures 10, 11, 12).

## 4 Discussion

In the present pilot study, the performance of (A) screw‐retained monolithic hybrid abutment crowns made of lithium disilicate and (B) adhesively cemented single‐tooth crowns on monolithic hybrid abutments made of lithium disilicate was observed over a clinical period of 3 years. A split‐mouth design was used for this study to enable a direct comparison of the two therapy alternatives, taking patient‐specific factors into account. The advantage here is that both types of restoration have the same preconditions (e.g., subjective demands on the restorations) and are exposed to the same external influences (e.g., chewing force, wear behavior). This minimized the bias caused due to patient specific parameters [48, 49].

After analysis of the present study results, the first hypothesis, which predicted no difference between the objective clinical evaluation criteria between the two types of restorations, was confirmed. No differences were found in the 3‐year survival and complication rates with regard to technical and biological criteria, as well as the evaluation of the restorations with the Functional Implant Prosthodontics Score (FIPS).

A survival rate of 100% was documented for both types of restoration in the present study, without the appearance of any technical or biological complications within the first 3 years. These results correspond to similar studies with clinical short‐term results, which also present very high survival rates for screw‐retained and cemented hybrid restorations. However, some of these studies show complications such as chipping of the ceramics, loosening of the abutment screws or slight peri‐implant inflammation. Complications were observed in both screw‐retained and cemented restorations [32, 33, 50, 51, 52]. In general, screw‐retained restorations are associated with more technical complications, while cemented restorations are more likely to have biological complications [35].

Chipping of ceramic restorations is mainly observed in veneered restorations [27]. The ceramic suprastructures used in the study were fabricated monolithically, so veneering was not required. At the same time, monolithic restorations show a higher fracture resistance, which can even resist the physiological forces in the posterior region and are also characterized by a wear‐ friendly relationship to the antagonistic tooth, compared to other ceramics [53].

In the present study, fractures of the ceramic superstructures themselves were also not observed. The restorations used in this study, consisting of a titanium adhesive base and a ceramic abutment superstructure, demonstrate an extremely stable connection to the implant, as the implant‐abutment connection consists purely of titanium, which is one material [54]. Fractures of the titanium adhesive base are rarely documented [55].

Complications that can occur in this area as a result of greater force are more likely focused on the comparatively weaker connection between the titanium base and ceramic abutment, which can lead to decementations of the abutment suprastructures that can then easily be recemented. Under in vitro conditions, this occurs more frequently with zirconium dioxide ceramics and significantly less with lithium disilicate [56]. In addition to the luting materials used for bonding the ceramic components to the titanium adhesive bases, the prosthetic design also plays a major role. In the present study, an adhesive luting material was used and the two composite components were conditioned for an optimal macro‐ and micromechanical bond. This is associated with a documented improved retention in the literature, resulting in a lack of decementation in this study [30, 57]. In addition, titanium bases with a sufficient height were used to provide optimum support for the ceramic superstructures. The prosthetic height of the titanium bases in particular has a significant influence on the fracture strength of the prosthetic restorations under in vitro conditions. The more the ceramic component is supported by the titanium base, the higher the fracture strength [56, 58] and the required removal forces [59]. This is a particularly an important factor for long crowns and excentric forces, which must be taken into account in the incisor region, for example in the present study, mainly posterior crowns were observed, which is why this appears to be less relevant for the technical complication rate. The use of lithium disilicate as a crown material is also advantageous compared to other ceramic materials [30, 56, 60].

Furthermore, fractures of the abutment screws are also reported in this context [61, 62].

In general, technical complications are often attributed to screw‐retained restorations in the literature [35]. However, the problem of screw loosening in particular is considered the most common technical complication for both types of restorations, cemented and screw‐retained [38, 40, 63]. For screw‐retained restorations, the lack of integrity of the restorations can lead to an uneven distribution of masticatory forces [21, 22]. At the same time, studies on cemented restorations also show that, for example, non‐axial loading of the restoration or parafunctions lead to similarly high loosening rates of the abutment screws [64, 65, 66]. Screw‐retained restoration offer the advantage of retrievability, as access to the screw channel is already available and allows the abutment screw to be exchanged quickly and easily. Cemented restorations are more challenging as the replacement of the abutment screw is associated with increased effort and destruction of the integrity of the crown. The advantage of cemented restorations in terms of a homogeneous occlusal surface is lost and/or the restoration has to be refabricated [35, 41]. These aspects in particular are interesting to consider due to the split‐mouth study design, as very similar forces apply to both types of restoration. However, no loosening was observed in any of the restorations in the present study. This can also be explained by the high fracture resistance of the ceramics when adequate parameters are used [67].

With regard to biological complications, the peri‐implant and transmucosal tissue plays a major role. Remaining cement residues in the sulcus, subgingival restoration margins or irritation of the soft tissue by material surfaces can be the reason for this [35, 36].

By using hybrid abutments, the restorations can be customized in their emergence profile, which has been proven to lead to a better biological reaction of the surrounding hard and soft tissue [16]. The position of the cement line in cemented restorations can be influenced and positioned further iso‐ or supragingivally in non‐visible areas.

Remaining cement residue in the sulcus can also lead to peri‐implant inflammation. Their complete removal is therefore essential and must be carried out carefully, which requires an iatrogenic factor [3, 68, 69]. The positive results in the following study are probably also due to the fact that all restorations were cemented by the same dentist with a correspondingly high level of practical and professional experience in the field of implantology.

Finally, with suitable preparation, lithium disilicate also exhibits very biocompatible behavior with the surrounding tissue, which is comparable with other ceramics and titanium [70, 71].

However, it is generally very difficult to draw a correct conclusion about the complication rate of both restorations due to the small number of test subjects in this pilot study. In order to be able to make meaningful conclusions for clinical use, the number of test subjects would have to be significantly increased [33]. The split‐mouth design used could represent a potential problem during recruitment, as it requires restorations in contralateral sides of the jaw at the same tooth positions [72].

Another possible reason for the lack of biological and technical complications in the present study is the relatively short observation period of the restorations of only 3 years in situ. This leads to the suggestion that the observation period used here could simply be too short before a complication could develop. Studies that have observed single‐tooth crowns made of lithium disilicate show high survival rates after 5 and 10 years, but various complications have been reported [26, 27, 73, 74, 75]. This leads to the conclusion that for an evaluation of both types of restorations with regard to their clinical use, further studies are necessary to test the materials for their clinical long‐term durability.

The second hypothesis of the study, which predicted no differences between the two types of restoration in terms of subjective, patient‐specific quality of life, can also be confirmed at least proportionately.

Both, patient satisfaction and oral health‐related quality of life, were rated as very high by the patients at the 36‐month follow‐up visit. In the OHIP evaluation, a significant improvement was already apparent after the restorations were cemented.

This may initially be due to the restoration of the single‐tooth gaps and the associated closing of the gaps in the dentition. Both subjective evaluation tools documented the subjective satisfaction of the patients even before the single‐tooth gaps were restored with implants or before the implants were inserted. Due to the missing teeth and corresponding tooth gaps, this was lower than at the later examination times when the gaps were closed. Although it is not absolutely necessary to restore individual tooth gaps in periodontally sufficient dentitions and chewing ability is demonstrably not impaired, the results show that other factors also have an important influence on the patients' evaluation of the dentures.

The limitation at this point, is that no direct comparison of the two restoration groups is possible due to the lack of a separate evaluation of both restorations. While the split‐mouth study design offers great advantages with regard to the objective evaluation of the restorations due to the reduction of bias caused by external factors, it represents a limitation here [48, 72]. In order to obtain a separate evaluation of the individual restoration types by the patient, the patients would need to be asked to complete both the VAS and the OHIP questionnaire for both restorations separately. For small examinations such as completing the VAS, this would be perfectly acceptable and easy to carry out. For more time‐consuming steps, this could be a limiting factor in patient recruitment. As a separate subjective assessment was not performed in the study, a direct comparison of the two types of restoration cannot be made. However, the very good results of patient satisfaction and OHIP suggest that there would be no major differences.

In the subjective evaluation of patient satisfaction with regard to esthetic appearance, as many as 8 out of 9 patients gave the best possible rating. Only one patient rated this as 8, but after consultation with the patient, this was due to a factor not directly related to the implant‐prosthetic restoration.

Due to their high translucency, from an esthetic point of view, lithium dislicate is an extremely popular and successful material for restoring teeth and implants [25]. As the same material was used for both restorations, the differences in the two restorations are probably not even visible to the patient. Due to the ceramic hybrid abutments in both cases, the soft tissue was also optimally conditioned and a gray shimmering through of titanium was covered [76, 77]. The only difference between the two restorations is the directly sealed screw channel with a tooth‐colored composite in the screw‐retained restorations. This may be visible, but as the majority of the restorations were located in the posterior region, it was probably not even noticed by the patient. From a functional point of view, both restorations are probably too similar for the patient to notice any difference.

## 5 Conclusion

Considering the limitations of the present pilot study, it can be concluded that over a clinical observation period of 3 years the restoration of single implants with screw‐retained monolithic hybrid abutment crowns and the restoration with monolithic hybrid abutments and adhesively bonded monolithic single‐tooth crowns are both sufficient therapy options and be evaluated as successful and satisfactory for both objective and subjective evaluation criteria without any superiority for one of both types of restoration.

## Author Contributions

**Michael Naumann:** conceptualization, investigation, methodology, data curation, writing – review and editing, project administration, funding acquisition. **Arndt Happe:** conceptualization, methodology, formal analysis, writing – review and editing, project administration. **Agnes Holtkamp:** writing – review and editing. **Sarah M. Blender:** formal analysis, data curation, writing – original draft preparation. All authors have read and agreed to the published version of the manuscript.

## Ethics Statement

The study was conducted in accordance with the Declaration of Helsinki, the German medical device law, ISO14155 Clinical Investigations of medical devices and good clinical practice and approved by the Institutional Review Board (or Ethic Committee) of the University of Ulm (approval number: 193/13, 2013‐07‐29). Also, the study was registered at the German Clinical Trials Register (Deutsches Register Klinischer Studien, ID: DRKS00005680).

## Consent

Informed consent was obtained from all subjects involved in the study.

## Conflicts of Interest

The authors declare no conflicts of interest.

## Data Availability

The data that support the findings of this study are available from the corresponding author upon reasonable request.
